# Correction: Determinants of Covid-19 vaccination: Evidence from the US pulse survey

**DOI:** 10.1371/journal.pgph.0002425

**Published:** 2023-09-18

**Authors:** The PLOS Global Public Health Staff

## Notice of republication

This article was republished on July 20, 2023, to replace the incorrect editor (“R&R PLOS”) with the correct editor (Collins Otieno Asweto). The publisher apologizes for the errors. Please download this article again to view the correct version. The originally published, uncorrected article and the republished, corrected articles are provided here for reference.

## Supporting information

S1 File Originally published, uncorrected article. (PDF)

S2 File Republished, corrected article. (PDF)
