# Supplementary material for: Correction: Determinants of Covid-19 vaccination: Evidence from the US pulse survey
Source: PLOS Glob Public Health. 2023 Sep 18;3(9):e0002425. doi: 10.1371/journal.pgph.0002425 (PMC10506702; doi:10.1371/journal.pgph.0002425)
Supplement: S2 File — (PDF) [file pgph.0002425.s002.pdf]

PLOS GLOBAL PUBLIC HEALTH

RESEARCH ARTICLE

Determinants of Covid-19 vaccination: Evidence from the US pulse survey

Amit Roy[1,2]*

1 Department of Economics, Shahjalal University of Science and Technology, Sylhet, Bangladesh,
2 Department of Economics, The New School for Social Research, New York, New York, United States of America

* amit-eco@sust.edu; roya221@newschool.edu

Abstract

The Covid-19 disease is resurging across the United States and vaccine hesitancy remains a major obstacle to reaching the expected threshold for herd immunity. Using the nationally representative cross sectional Household Pulse Survey (HPS) Data published by the U.S. Census Bureau, this study identified demographic, socio-economic, and medical-psychological determinants of Covid-19 vaccination. Results revealed significant differences in Covid-19 vaccine uptake due to age, sex, sexual orientation, race or ethnicity, marital status, education, income, employment form, housing and living condition, physical illness, mental illness, Covid-19 illness, distrust of vaccines and beliefs about the efficacy of vaccines. Government policymakers need to be cognizant of these determinants of vaccine hesitancy when formulating policies to increase vaccine uptake and control the COVID-19 pandemic. The findings of this study suggest that segmented solutions to reach vulnerable groups like racial minorities and homeless people are needed to win the trust and optimize vaccine uptake.

Check for updates

OPEN ACCESS

Citation: Roy A (2023) Determinants of Covid-19 vaccination: Evidence from the US pulse survey. PLOS Glob Public Health 3(5): e0001927. https://doi.org/10.1371/journal.pgph.0001927

Editor: Collins Otieno Asweto, University of Embu, KENYA

Received: January 10, 2023

Accepted: April 24, 2023

Published: May 18, 2023

Copyright: © 2023 Amit Roy. This is an open access article distributed under the terms of the Creative Commons Attribution License, which permits unrestricted use, distribution, and reproduction in any medium, provided the original author and source are credited.

Data Availability Statement: Data are readily available for download from https://www.census.gov/data/experimental-data-products/household-pulse-survey.html.

Funding: The authors received no specific funding for this work.

Competing interests: The authors have declared that no competing interests exist.

Introduction

The Coronavirus disease 2019 (COVID-19), caused by Severe Acute Respiratory Syndrome Coronavirus-2 (SARS-CoV-2), is still spreading around the world. Globally, COVID-19 has infected close to 659 million people with more than 6.7 million deaths affecting 228 countries and territories as of December 20, 2022 [1]. In the United States alone, the number of COVID-19 cases exceeds 102 million with more than 1.1 million deaths [1]. The spread of COVID-19 infection and recurring surges in the number of new cases and deaths have caused catastrophic social and economic impacts around the world [2]. During the pandemic, governments restricted travelers from other countries and imposed nationwide lockdowns which affected the quality of life for millions. The social, economic, and health tolls of COVID-19 in the United States have been among the highest in the world [3].

Vaccination is one of the most successful and cost-effective interventions known to improve health outcomes [4]. Vaccines provide prophylaxis against infectious diseases and are responsible for saving a significant number of lives over more than two centuries [5].

Vaccination thus has become increasingly important for responses to the COVID-19 pandemic and conquering the pandemic eventually. Vaccines designed to protect against COVID-19 were developed in an unprecedentedly rapid period of time. So far, four COVID-19 vaccines are approved or authorized in the United States, namely, Pfizer-BioNTech, Moderna, Novavax, and Johnson & Johnson's Janssen. COVID-19 vaccines available in the United States are effective at protecting people from getting seriously ill, being hospitalized, and dying [6]. As of December 20, 2022, at least 262,908,216 people, that is, 79% of the US population have received at least one dose of the COVID-19 vaccine while 224,113,439 people, that is, 68% of the population are fully vaccinated [7]. Additionally, 108,806,974 people, that is, 33% of the US population have received a booster dose [7].

Despite the availability of safe and efficacious COVID-19 vaccines, a significant proportion of the American public remains unvaccinated and does not appear to be immediately interested in receiving the vaccine which is large enough to threaten to achieve herd immunity, decrease the rate of hospitalization and lower mortality [8]. Vaccine hesitancy poses dangers to both the individual and his or her community, since exposure to a contagious disease places the person at risk, and individuals are far more likely to spread the disease to others if they do not get vaccinated [9]. Therefore, vaccine acceptance and hesitancy among the general population play an important role in successfully controlling the COVID-19 pandemic.

Vaccine hesitancy refers to the delay in acceptance, reluctance, or refusal of vaccination or having one child vaccinated despite the availability of vaccination services [10]. It is a complex, context-specific, and rapidly changing global problem that varies across time, place, and vaccines [4]. Vaccine hesitancy is a behavior influenced by a number of factors including issues of confidence (do not trust the vaccine or provider), complacency (do not perceive a need for a vaccine, do not value the vaccine), and convenience (access) [11].

Moreover, several factors fueled the acceptance or refusal of COVID-19 vaccines including demographic factors (like age, gender, ethnicity, and marital status) [12], socioeconomic factors (like education, employment status, income, and wealth) [13], biomedical factors (like chronic physical illness, mental illness), geography, religiosity, culture, and political leaning [14] etc. For instance, males, married, older adults, Asians, and graduate degree holders are more likely to accept the vaccine in the United States [15]. On the other hand, being younger, having lost of income during the pandemic, those who had children at home, being rural dwellers, have low confidence in the COVID-19 vaccine and the health service response during the pandemic, have the worse perception of government measures, and perception of the information provided as inconsistent and contradictory are associated with both refusal and delay [16]. Additionally, black Americans and those with the least schooling were also less likely to receive vaccines for themselves or the people in their care in the United States [17]. Current data show a disproportionate burden of COVID-19 infections and deaths among racial and ethnic minority communities in the United States as Black Americans are three times more likely than White Americans to contract Covid-19 [18].

Given the high prevalence of COVID-19 vaccine hesitancy, evidence-based policy measures are needed across the United States to convert vaccines into vaccinations, protect the most vulnerable populations, reopen social and economic life, and potentially achieve herd immunity [8]. The factors of vaccine hesitancy or refusal are crucial dimensions that are required to be understood in order to design appropriate interventions [19]. Against this backdrop, the aim of this study is to explore the determinants of COVID-19 vaccination and to provide recommendations to increase the acceptance and uptake of COVID-19 vaccines in the United States. The novel contribution of this study is threefold. Firstly, it incorporates the role of sexual orientation as a determinant of COVID-19 vaccine uptake focusing on the LGBTQIA+ (lesbian, gay, bisexual, transgender, queer, intersex and asexual) community. Secondly, it incorporates

Table 1. Summary statistics.

Variables and Categories	Frequency (Percentage)
**Observations**	69,114
Demographic Factors	
Age: Mean	53.14 Years
Sex: Male	41,522(60.08%)
Female	27,592(39.92%)
Sexual Orientation: Straight	61,238(88.60%)
LGBTQIA+	7,876 (11.40%)
Race: White	51,691(74.79%)
Black	6,454 (9.34%)
Hispanic	5,103 (7.38%)
Asian	3,354 (4.85%)
Mixed	2,512 (3.63%)
Marital Status: Married	40,036(58.68%)
Widowed	3,872 (5.67%)
Divorced	10,310 (15.11%)
Separated	1,214 (1.78%)
Single	12,801(18.76%)
Socio-Economic Factors	
Education: Less than high school	411 (0.59%)
Some high school	936 (1.35%)
High school graduate or equivalent	7,857(11.37%)
Associate's degree (e.g., AA, AS)	14,596 (21.12%)
Some college, but degree not received	7,508(10.86%)
Bachelor's degree	20,075(29.05%)
Graduate degree (master's, doctorate)	17,731(25.65%)
Total Household Income: Less than $25,000	5,698 (10.46%)
Between $25,000 - $34,999	4,600 (8.44%)
Between $35,000 - $49,999	5,805 (10.66%)
Between $50,000 - $74,999	9,330 (17.13%)
Between $75,000 - $99,999	7,830 (14.37%)
Between $100,000 - $149,999	10,117(18.57%)
Between $150,000 - $199,999	4,980 (9.14%)
$200,000+	6,117 (11.23%)
Doing Inperson Work	34,918(50.52%)
Housing and Living Condition: One-family house detached from any other house	1,781(3.08%)
A one-family house attached to one or more houses	6,731(11.65%)
A building with two apartments	41,348 (71.56%)
A building with 3 or 4 apartments	4,628 (8.01%)
A building with 5 or more apartments	1,737 (3.01%)
A mobile home, boat, van, etc.	1,269 (2.20%)
Homeless	284(0.49%)
Received Pandemic Stimulus Benefits	14,198 (20.54%)
Medical-Psychological Factors	
Have Chronic Physical Illness	14,537 (24.01%)
Have Mental Illness	9,122 (13.20%)
Detected Covid Before	23112(33.44%)
Have Health Insurance	50,711 (73.37%)

(*Continued*)

Table 1. (Continued)

Variables and Categories	Frequency (Percentage)
Received Covid Vaccine: No	8,788 (12.72%)
Yes	60,326 (87.28%)
I don't trust Covid vaccine	8,116 (11.74%)
Covid vaccine wouldn't protect me	6,641 (9.61%)
I don't believe I need a COVID-19 vaccine	2,125 (3.07%)

https://doi.org/10.1371/journal.pgph.0001927.t001

the role of the pandemic stimulus package as a determinant of COVID-19 vaccination. Finally, it introduces Probit models to find out the determinants of COVID-19 vaccine uptake in the United States which has never been done before.

Materials and methods

Data

This paper employs the Household Pulse Survey (HPS) Data published by the U.S. Census Bureau. The HPS is a biweekly cross-sectional survey of US households designed to deploy quickly and efficiently collecting data to measure household experiences during the coronavirus pandemic and recovery. The HPS is a 20-minute online survey studying how the coronavirus pandemic and other emergent issues are impacting American households across the country from a socioeconomic and health perspective. Sampling was drawn from the Census Bureau Master Address File and the Census Bureau Contract Frame, containing approximately 140 million housing units with matched phone or email contacts. Households were contacted by email and text message, and data were collected via the online survey. The HPS is a continuing project, and additional phases have been scheduled. For this analysis, we use the Phase 3.5: June 1, 2022 –August 8, 2022 dataset [20].

Statistical method

Probit regression is used to model dichotomous or binary outcome variables. The purpose of the model is to estimate the probability that an observation with particular characteristics will fall into a specific one of the categories. Here, the response variable Y is binary, that is it can have only two possible outcomes which we will denote as 1 and 0. Those who received at least one dose of the Covid-19 vaccine are designated as 1 and those who did not receive any dose of the Covid-19 vaccine are designated as 0. The probability of being vaccinated ($P(Y = 1)$) is here a function of demographic, socio-economic and medical-psychological covariates (X^T).

$$P(Y = 1) = \phi(X^T\beta)$$

$$P(Y = 0) = 1 - \phi(X^T\beta)$$

where P is the probability and ϕ is the cumulative distribution function of the standard normal distribution. The parameters, β, are estimated by the maximum likelihood estimator. X^T includes *demographic factors* like age, sex at birth (male or female), sexual orientation (straight or LGBTQIA+), race (White, Black, Hispanic, Asian or Mixed), marital status (married, widowed, divorced, separated or single), *socio-economic factors* like level of education, total household income, the form of employment (inperson or online), housing and living conditions and whether or not received pandemic stimulus benefit as well as *medical-psychological factors* like chronic physical illness, mental illness, Covid-19 illness, health insurance, trust on Covid-19

vaccine (I don't trust Covid vaccine) and beliefs about COVID-19 vaccine (Covid vaccine wouldn't protect me or I don't believe I need a COVID-19 vaccine).

Results and discussion

Summary statistics of the variables

Table 1 presents the frequency and percentage statistics of the variables under the study. The analytical sample consists of 69,114 participants of whom 60,326 (87.28%) received at least one dose of the COVID-19 vaccine and 8,788 (12.72%) did not receive the COVID-19 vaccine. COVID-19 vaccine hesitancy is measured by the response from the survey participants who were not fully vaccinated and did not express definite intentions of getting the COVID-19 vaccine and answered the following questions: 1) I don't trust COVID-19 vaccines; 2). I don't know if a COVID-19 vaccine will protect me; 3). I don't believe I need a COVID-19 vaccine. Answers to the questions are coded as 1 for yes and 0 for no. The detailed analysis of summary statistics is exhausted for brevity.

Result of probit analysis

Table 2 reports the results of the Probit models analysed in STATA software. The null hypothesis of the Wald chi-square test is that at least one of the predictors' regression coefficients is not equal to zero. The Wald chi-square statistics with a p-value of 0.000 tells us that our models as a whole are statistically significant, that is, they fit significantly better than models with no predictors. Pseudo R-square reports the goodness of the fits of the models.

The coefficient of age is negative and the coefficient of age squared is positive which implies that there exists a U-shaped relationship between age and the predicted probability of Covid-19 vaccination. This means that an increase in age increases the predicted probability of Covid-19 vaccination after a threshold point of age. This is because of the fact that as for age differences, confidence was found to decrease with age whereas perceived needs were found to increase with age [12]. These age differences are consistent with prior research on vaccine hesitancy as younger age cohorts are more likely to be healthy and have fewer underlying health conditions, as a result, they may not perceive the need of taking the vaccine. On the other hand, older age people are more likely to suffer from chronic diseases and more likely to be vaccinated.

The coefficient of sex is positive which indicates that females are more likely to be vaccinated than the reference group of males at <0.01 level of significance. These findings are generally in line with evidence revealed in prior research. Compared to men, women, in general, are more likely to engage in preventive medicine and use health services [21]. During the COVID-19 pandemic, women are more likely to perceive it as a serious health problem and abide by public health restrictions than men. The coefficient of sexual orientation is positive which implies that LGBTQIA+ people are more likely to be vaccinated than the reference group of straights at <0.01 level of significance. This may be the case due to the fact that LGBTQIA+ people are more prone to infectious diseases like HIV and are more likely to believe in the safety and protection of vaccines than heterosexual adults.

The coefficients of race reveal that Black Americans are 14% less likely to get Covid-19 vaccination than White Americans at <0.01 level of significance. Moreover, people of mixed race are 3.4% less likely to get Covid-19 vaccination than White Americans at <0.10 level of significance. However, Hispanic and people of Asian origin are more likely to be vaccinated than the reference group of White Americans <0.01 level of significance. Institutional racism and historical inequities in health care may also play a role in vaccine hesitancy among African Americans and other people of color [22].

Table 2. Result of probit models. Dependent variable is the Covid-19 vaccination status (1 = vaccinated, 0 = unvaccinated).

Variables	(1)	(2)	(3)
Demographic Factors			
Age	-0.013**	-0.011**	-0.009**
	(0.006)	(0.005)	(0.005)
Age-squared	0.001***	0.001***	0.001***
	(0.000)	(0.000)	(0.000)
Sex: Male	Ref	Ref	Ref
Female	0.021***	0.049***	0.068***
	(0.006)	(0.011)	(0.019)
Sexual Orientation: Straight	Ref	Ref	Ref
LGBTQIA+	0.087***	0.191***	0.264***
	(0.028)	(0.037)	(0.034)
Race: White	Ref	Ref	Ref
Black	-0.140***	-0.048***	-0.025***
	(0.047)	(0.011)	(0.004)
Hispanic	0.356***	0.492***	0.529***
	(0.090)	(0.074)	(0.065)
Asian	0.034***	0.123***	0.122***
	(0.008)	(0.037)	(0.033)
Mixed	-0.122*	-0.080*	-0.086*
	(0.067)	(0.041)	(0.045)
Marital Status: Married	Ref	Ref	Ref
Widowed	-0.091**	-0.127**	-0.118**
	(0.044)	(0.050)	(0.046)
Divorced	-0.040***	-0.091***	-0.072***
	(0.010)	(0.030)	(0.027)
Separated	-0.113	-0.111	-0.176
	(0.093)	(0.072)	(0.091)
Single	0.119***	0.081***	0.103***
	(0.042)	(0.019)	(0.028)
Socio-Economic Factors			
Education: Less than high school	Ref	Ref	Ref
Some high school	0.190**	0.091**	0.080**
	(0.073)	(0.031)	(0.032)
High school graduate or equivalent	0.095**	0.162**	0.087**
	(0.046)	(0.081)	(0.042)
Associate's degree (e.g., AA, AS)	0.169**	0.292**	0.201**
	(0.065)	(0.115)	(0.096)
Some college, but degree not received	0.258***	0.369***	0.269***
	(0.068)	(0.117)	(0.082)
Bachelor's degree	0.498***	0.670***	0.563***
	(0.167)	(0.116)	(0.107)
Graduate degree (master's, doctorate)	0.508***	0.738***	0.671***
	(0.168)	(0.118)	(0.108)
Total Household Income: Less than $25,000	Ref	Ref	Ref
Between $25,000 - $34,999	0.119***	0.186***	0.152***
	(0.034)	(0.041)	(0.036)
Between $35,000 - $49,999	0.254***	0.289***	0.294***

(*Continued*)

Table 2. (Continued)

Variables	(1)	(2)	(3)
	(0.052)	(0.039)	(0.035)
Between $50,000 - $74,999	0.312***	0.384***	0.372***
	(0.050)	(0.037)	(0.034)
Between $75,000 - $99,999	0.350***	0.403***	0.404***
	(0.054)	(0.041)	(0.037)
Between $100,000 - $149,999	0.439***	0.493***	0.533***
	(0.055)	(0.041)	(0.037)
Between $150,000 - $199,999	0.456***	0.563***	0.631***
	(0.068)	(0.052)	(0.047)
$200,000+	0.479***	0.640***	0.670***
	(0.069)	(0.053)	(0.048)
Doing Inperson Work: No	Ref	Ref	Ref
Yes	0.071**	0.045**	0.045**
	(0.030)	(0.023)	(0.020)
Housing and Living Condition: One-family house detached from any other house	Ref	Ref	Ref
A one-family house attached to one or more houses	0.104***	0.175***	0.184***
	(0.039)	(0.050)	(0.043)
A building with two apartments	0.118***	0.218***	0.272***
	(0.042)	(0.061)	(0.053)
A building with 3 or 4 apartments	0.099**	0.151**	0.168**
	(0.046)	(0.076)	(0.068)
A building with 5 or more apartments	0.107**	0.180**	0.292***
	(0.037)	(0.071)	(0.064)
A mobile home, boat, van, etc.	0.193**	0.396***	0.382***
	(0.077)	(0.058)	(0.050)
Homeless	-0.223***	-0.341***	-0.109***
	(0.080)	(0.125)	(0.023)
Received Pandemic Stimulus Benefits: No	Ref	Ref	Ref
Yes	0.020**	0.048**	0.034**
	(0.009)	(0.024)	(0.011)
Medical-Psychological Factors			
Have Chronic Physical Illness: No	Ref	Ref	Ref
Yes	0.124***	0.138***	0.180***
	(0.038)	(0.029)	(0.029)
Have Mental Illness: No	Ref	Ref	Ref
Yes	0.238***	0.271***	0.280***
	(0.034)	(0.026)	(0.023)
Detected Covid Before: No	Ref	Ref	Ref
Yes	0.480***	0.476***	0.529***
	(0.033)	(0.026)	(0.023)
Have Health Insurance: No	Ref	Ref	Ref
Yes	0.001	0.032	0.012
	(0.041)	(0.031)	(0.028)
I don't trust Covid vaccine	-.601***	-	-
	(0.047)	-	-
Covid vaccine wouldn't protect me	-	-0.633***	-
	-	(0.058)	-

(*Continued*)

Table 2. (Continued)

Variables	(1)	(2)	(3)
I don't believe I need a COVID-19 vaccine	-	-	-0.482***
	-	-	(0.073)
Constant	0.993***	0.786***	0.865***
	(0.246)	(0.181)	(0.163)
Observations	69,114	53,741	53,741
Wald x^2 (chi-square)	7748.22***	5239.74***	4465.08***
(P value)	(0.000)	(0.000)	(0.000)
Pseudo R^2	0.7372	0.6110	0.5644

Notes: (1) Robust standard errors in parentheses.

(2) ***, ** and * denotes p-value <0.01, <0.05, and p<0.1 respectively.

(3) Ref means reference group.

https://doi.org/10.1371/journal.pgph.0001927.t002

The coefficients of marital status disclose that widowed and divorced Americans are less likely to get Covid-19 vaccination than married Americans at the conventional level of significance. This may be due to the fact that married people are more concerned about their children health and feel the necessity of being vaccinated in order to protect their offspring. However, bachelor Americans are 12% more likely to get Covid-19 vaccination than married Americans at <0.01 level of significance.

The coefficients of educational status expose that people with a formal educational degree are more likely to get Covid-19 vaccination than those with less than high school or no formal degree at the conventional level of significance. It is consistent with the prior research that educational levels are positively related to safety concerns and thus people with higher education levels are more likely to be vaccinated [23].

The coefficients of household income imply that households with higher income are more likely to get Covid-19 vaccination than those with lower income at the conventional level of significance. People with high income are more concerned about their health care and physical fitness and have better access to medications and vaccines than the low-income people. Moreover, this study finds that persons doing inperson work are 7% more likely to get Covid-19 vaccination than those doing work virtually at <0.05 level of significance. Persons working outside inperson are more vulnerable to catching Covid-19 than persons doing online work from home and thus they have higher propensity to be vaccinated.

The coefficients of housing and living conditions indicate that homeless people are 22.3% less likely to get Covid-19 vaccination than those living in one-family house detached from any other house at <0.01 level of significance. Vaccine access for persons experiencing homelessness can be enhanced by using multiple strategies, including pop-up vaccination clinics in convenient locations, mobile clinics in partnership with trusted providers, and street outreach teams. Additionally, households that received pandemic stimulus benefits from the government are 2% more likely to get Covid-19 vaccination than those who did not receive any pandemic stimulus benefits from the government at <0.05 level of significance. Fiscal stimulus provides incentives to the people and motivated them to take vaccines in order to get future monetary benefits.

Medical and psychological factors reveal that persons with a chronic physical illness like diabetes, asthma, arthritis, chronic heart, lung or kidney disease are 12% more likely to get Covid-19 vaccination than those who did not any chronic physical illness at <0.01 level of

significance. Moreover, persons with a mental illness are 28% more likely to get Covid-19 vaccination than those who did not any mental illness at <0.01 level of significance. Respondents with chronic respiratory disease and those with autoimmune diseases were more likely to want to be vaccinated to protect themselves from COVID-19 [24]. Persons who tested Covid-19 positive before are 48% more likely to get Covid-19 vaccination than those who did not detect such illness at <0.01 level of significance. Though prior Covid-19 infection had offered protection against reinfection before the participants were vaccinated, this protection had waned. Vaccination provided increased, longer-lasting protection to previously infected participants, and it gave them greater than 90% protection against reinfection more than 18 months after the primary infection [25]. However, this paper finds no statistically significant relationship between Covid-19 vaccination and health insurance coverage which implies that since Covid-19 vaccines are publicly provided free of charge, health insurance does not matter.

Moreover, people who don't have trust in the Covid-19 vaccine are 60% less likely to be vaccinated than those who have trust in the Covid-19 vaccine at <0.01 level of significance (Model 1). Trust is a core predictor, with distrust in vaccines raising vaccine hesitancy. The most given reasons for the general lack of trust in vaccines are doubts about the efficiency of the vaccine and concerns about the side effects of the vaccine. People who believe that the Covid-19 vaccine wouldn't protect them are 63% less likely to be vaccinated than those who do not have such belief at <0.01 level of significance (Model 2). People who believe that they don't need the Covid-19 vaccine are 48% less likely to be vaccinated than those who do not have such belief at <0.01 level of significance (Model 3). What is more, COVID-19 is not only a pandemic, but an *infodemic* [26]. The growth in internet use and reliance on social media sources such as YouTube, Facebook, Twitter, and TikTok has changed the landscape of information gathering [27]. Low trust in vaccines and anti-vax beliefs are associated with a greater reliance on social media for health information generation [28]. Thus, along with removing barriers to vaccination and educational campaigns about vaccine safety and the societal benefits of high vaccination uptake, mandates could be introduced for increasing vaccination uptake to the required levels.

Conclusion

The Covid-19 disease is resurging across the United States and vaccine hesitancy remains a major obstacle to reaching the expected threshold for herd immunity. Using the nationally representative Household Pulse Survey (HPS) Data published by the U.S. Census Bureau, this study identified demographic, socio-economic and medical-psychological determinants of Covid-19 vaccination. Results revealed significant differences in Covid-19 vaccine uptake due to age, sex, sexual orientation, race or ethnicity, marital status, education, income, employment form, housing and living condition, physical illness, mental illness, Covid-19 illness, distrust of vaccines and beliefs about the efficacy of vaccines. Government policymakers need to be cognizant of these determinants of vaccine hesitancy when formulating policies to increase vaccine uptake and control the COVID-19 pandemic. These findings further suggest that much more work needs to be done to enhance the uptake of the vaccine, most importantly, public health measures must be aligned with the social realities of America experienced by racial minorities and homeless people. Segmented solutions to reach vulnerable groups are needed to win the trust and optimize vaccine uptake.

However, this study is not without limitations and invites extensions. Due to the unavailability of data, the study could not incorporate the sources of vaccine distrust like the effect of past vaccine or medication intake on the decision of taking a new vaccine intake, religious

belief and whether the brand of vaccine supplier matters. This paves the way for further research in this area.

Author Contributions

Conceptualization: Amit Roy.

Data curation: Amit Roy.

Formal analysis: Amit Roy.

Methodology: Amit Roy.

Software: Amit Roy.

Writing – original draft: Amit Roy.

Writing – review & editing: Amit Roy.

References

1. World Metrometer. COVID-19 Coronavirus Pandemic. 2022 Accessed on December 20, 2022. https://www.worldometers.info/coronavirus/?isci=010702
2. Belostecinic G, Mogos R, Popescu ML, Burlacu S, Radulescu CV, Bodislav AD, et al. Teleworking—An Economic and Social Impact during COVID-19 Pandemic: A Data Mining Analysis. International journal of environmental research and public health. 2022 Jan 3; 19(1):1–36. DOI: 10.3390/ijerph19010298
3. Khubchandani J, Sharma S, Price JH, Wiblishauser MJ, Sharma M, Webb FJ. COVID-19 vaccination hesitancy in the United States: a rapid national assessment. Journal of community health. 2021 Apr; 46 (2):270–7. https://doi.org/10.1007/s10900-020-00958-x PMID: 33389421
4. Eskola J, Duclos P, Schuster M, MacDonald NE. How to deal with vaccine hesitancy? Vaccine. 2015 Aug 14; 33(34):4215–7. https://doi.org/10.1016/j.vaccine.2015.04.043 PMID: 25896378
5. Moore R, Willis DE, Shah SK, Purvis RS, Shields X, McElfish PA. "The risk seems too high": thoughts and feelings about COVID-19 vaccination. International journal of environmental research and public health. 2021 Aug 17; 18(16):8690. https://doi.org/10.3390/ijerph18168690 PMID: 34444438
6. Mollalo A, Tatar M. Spatial modeling of COVID-19 vaccine hesitancy in the United States. International journal of environmental research and public health. 2021 Sep 8; 18(18):9488. https://doi.org/10.3390/ijerph18189488 PMID: 34574416
7. USA Fact. US COVID-19 cases and deaths by states. 2022. https://usafacts.org/visualizations/covid-vaccine-tracker-states
8. Tram KH, Saeed S, Bradley C, Fox B, Eshun-Wilson I, Mody A, et al. Deliberation, Dissent, and Distrust: Understanding Distinct Drivers of Coronavirus Disease 2019 Vaccine Hesitancy in the United States. Clinical infectious diseases. 2022 Apr 15; 74(8):1429–41. https://doi.org/10.1093/cid/ciab633 PMID: 34272559
9. Coustasse A, Kimble C, Maxik K. COVID-19 and vaccine hesitancy: a challenge the United States must overcome. The Journal of ambulatory care management. 2021 Jan 1; 44(1):71–5. https://doi.org/10.1097/JAC.0000000000000360 PMID: 33165121
10. Soares P, Rocha JV, Moniz M, Gama A, Laires PA, Pedro AR, et al. Factors associated with COVID-19 vaccine hesitancy. Vaccines. 2021 Mar 22; 9(3):300. https://doi.org/10.3390/vaccines10020281 https://doi.org/10.3390/vaccines9030300 PMID: 33810131
11. Troiano G, Nardi A. Vaccine hesitancy in the era of COVID-19. Public health. 2021 May 1; 194:245–51. https://doi.org/10.1016/j.puhe.2021.02.025 PMID: 33965796
12. Shih SF, Wagner AL, Masters NB, Prosser LA, Lu Y, Zikmund-Fisher BJ. Vaccine hesitancy and rejection of a vaccine for the novel coronavirus in the United States. Frontiers in immunology. 2021 Jun 14; 12:558270. https://doi.org/10.3389/fimmu.2021.558270 PMID: 34194418
13. Lee J, Huang Y. COVID-19 Vaccine Hesitancy: The Role of Socioeconomic Factors and Spatial Effects. Vaccines. 2022 Feb 24; 10(3):352. https://doi.org/10.3390/vaccines10030352 PMID: 35334984
14. Wu YY, Zhang W. Demographic disparities in COVID-19 vaccine hesitancy among US adults: Analysis of household pulse survey data from Jul 21 to Oct 11 in 2021. Vaccine. 2022 Dec 12; 40(52):7510–4. https://doi.org/10.1016/j.vaccine.2022.10.094

15. Malik AA, McFadden SM, Elharake J, Omer SB. Determinants of COVID-19 vaccine acceptance in the US. EClinicalMedicine. 2020 Sep 1; 26:100495. https://doi.org/10.1016/j.eclinm.2020.100495 PMID: 32838242
16. Aw J, Seng JJ, Seah SS, Low LL. COVID-19 vaccine hesitancy—A scoping review of literature in high-income countries. Vaccines. 2021 Aug 13; 9(8):900. https://doi.org/10.3390/vaccines9080900 PMID: 34452026
17. Viswanath K, Bekalu M, Dhawan D, Pinnamaneni R, Lang J, McLoud R. Individual and social determinants of COVID-19 vaccine uptake. BMC Public Health. 2021 Dec; 21(1):1–0. https://doi.org/10.1186/s12889-021-10862-1
18. Bunch L. A tale of two crises: addressing Covid-19 vaccine hesitancy as promoting racial justice. InHec Forum 2021 Jun (Vol. 33, No. 1, pp. 143–154). Springer Netherlands. DOI: 10.1007/s10730-021-09440-0
19. McElfish PA, Willis DE, Shah SK, Bryant-Moore K, Rojo MO, Selig JP. Sociodemographic determinants of COVID-19 vaccine hesitancy, fear of infection, and protection self-efficacy. Journal of primary care & community health. 2021 Aug; 12:21501327211040746. https://doi.org/10.1177/21501327211040746 PMID: 34427126
20. US Bureau of Census. Household Pulse Survey Data. 2022. https://www.census.gov/programs-surveys/household-pulse-survey/data.html
21. Lee BY, Mueller LE, Tilchin CG. A systems approach to vaccine decision making. Vaccine. 2017 Jan 20; 35: A36–42. https://doi.org/10.1016/j.vaccine.2016.11.033 PMID: 28017430
22. Golden, S. COVID-19 Vaccines and People of Color. 2022. https://www.hopkinsmedicine.org/health/conditions-and-diseases/coronavirus/covid19-vaccines-and-people-of-color
23. Klein NP. Added benefit of Covid-19 vaccination after previous infection. New England Journal of Medicine. 2022 Mar 31; 386(13):1278–9. https://doi.org/10.1056/NEJMe2201380 PMID: 35353966
24. Nikolovski J, Koldijk M, Weverling GJ, Spertus J, Turakhia M, Saxon L, et al. Factors indicating intention to vaccinate with a COVID-19 vaccine among older US adults. PloS one. 2021 May 24; 16(5): e0251963. https://doi.org/10.1371/journal.pone.0251963
25. Smith BA, Ricotta EE, Kwan JL, Evans NG. COVID-19 risk perception and vaccine acceptance in individuals with chronic disease. medRxiv. 2021 Mar 24:2021–03. doi: 10.1101/2021.03.17.21253760
26. Jennings W, Stoker G, Bunting H, Valgarðsson VO, Gaskell J, Devine D, et al. Lack of trust, conspiracy beliefs, and social media use predict COVID-19 vaccine hesitancy. Vaccines. 2021 Jun 3; 9(6):593. https://doi.org/10.3390/vaccines9060593 PMID: 34204971
27. Puri N, Coomes EA, Haghbayan H, Gunaratne K. Social media and vaccine hesitancy: new updates for the era of COVID-19 and globalized infectious diseases. Human vaccines & immunotherapeutics. 2020 Nov 1; 16(11):2586–93. https://doi.org/10.1080/21645515.2020.1780846 PMID: 32693678
28. Pertwee E, Simas C, Larson HJ. An epidemic of uncertainty: rumors, conspiracy theories and vaccine hesitancy. Nature medicine. 2022 Mar; 28(3):456–9. https://doi.org/10.1038/s41591-022-01728-z PMID: 35273403
